# Cirmtuzumab inhibits ibrutinib-resistant, Wnt5a-induced Rac1 activation and proliferation in mantle cell lymphoma

**DOI:** 10.18632/oncotarget.25340

**Published:** 2018-05-15

**Authors:** Jian Yu, Yun Chen, Liguang Chen, Ling Zhang, Laura Z. Rassenti, George F. Widhopf, Thomas J. Kipps

**Affiliations:** ^1^ Moores Cancer Center, University of California, San Diego, La Jolla, CA 92093, USA

**Keywords:** cirmtuzumab, ibrutinib, Wnt5a, ROR1, MCL

## Abstract

Cirmtuzumab may enhance the therapeutic activity of ibrutinib by inhibiting ROR1-dependent signaling pathway in patients with chronic lymphocytic leukemia (CLL). Mantle cell lymphoma (MCL) is B-cell malignancy that also expresses ROR1. In this study, we found that the plasma of patients with MCL had high levels of Wnt5a, a ROR1 ligand, that were comparable to those found in patients with CLL; in contrast Wnt5a was virtually undetectable in the plasma of age-matched healthy adults. We also found that Wnt5a induced Rac1 activation in the primary MCL cells. Cirmtuzumab, but not ibrutinib, could inhibit the capacity of Wnt5a to induce primary MCL cells to activate Rac1. Addition of exogenous Wnt5a *in vitro* significantly enhanced the numbers of MCL cell divisions and the proportion of dividing MCL cells entering S/G2 in MCL cells over time in the presence of CD154 and IL-4/10. Treatment of the MCL cells with cirmtuzumab, but not ibrutinib, blocked Wnt5a-enhanced proliferation of MCL cells. This study indicates that cirmtuzumab and ibrutinib may have complementary activity in the treatment of patients with MCL.

## INTRODUCTION

Recent studies have demonstrated that Wnt5a could induce activation of Rac1 in leukemia cells of patients with CLL via a ROR1-dependent pathway [1], which could not be inhibited by ibrutinib [2]. Wnt5a could induce Rac1 activation to enhance proliferation and survival of ROR1-expressing CLL cells, even when treated with ibrutinib at concentrations that were effective in inhibiting BTK and B-cell-receptor (BCR) signaling [2]. Such Wnt5a-induced Rac1 activation could be blocked by cirmtuzumab (UC-961), a humanized anti-ROR1 mAb, which is undergoing clinical evaluation in patients with CLL [3]. Finally, treatment of CLL cells with cirmtuzumab and ibrutinib was significantly more effective than treatment with either agent alone in blocking CLL proliferation and in clearing leukemia cells *in vivo* [2], suggesting that cirmtuzumab may enhance the therapeutic activity of ibrutinib in patients with CLL.

Mantle cell lymphoma (MCL) is B cell malignancy that also expresses ROR1 [4]. However, it is not known whether Wnt5a can induce Rac1 activation in MCL or whether such activation is affected by treatment with either ibrutinib or cirmtuzumab. This study demonstrates that Wnt5a can induce Rac1 activation, which leads to enhanced proliferation of primary MCL cells via a ROR1-dependent signaling-pathway that can be blocked by cirmtuzumab. On the other hand, this signaling-pathway cannot be blocked by ibrutinib, even at concentrations that completely inhibit BTK or BCR-signaling.

## RESULTS

We examined primary neoplastic cells of patients with MCL who had circulating leukemia cells for Wnt5a-induced, ROR1-dependent activation of Rac1. The neoplastic B cells of each examined patient (*n* = 8) each had the t(11;14) cytogenetic abnormality and the immune phenotype characteristic of MCL (Supplementary Figure 1).

**Figure 1:**
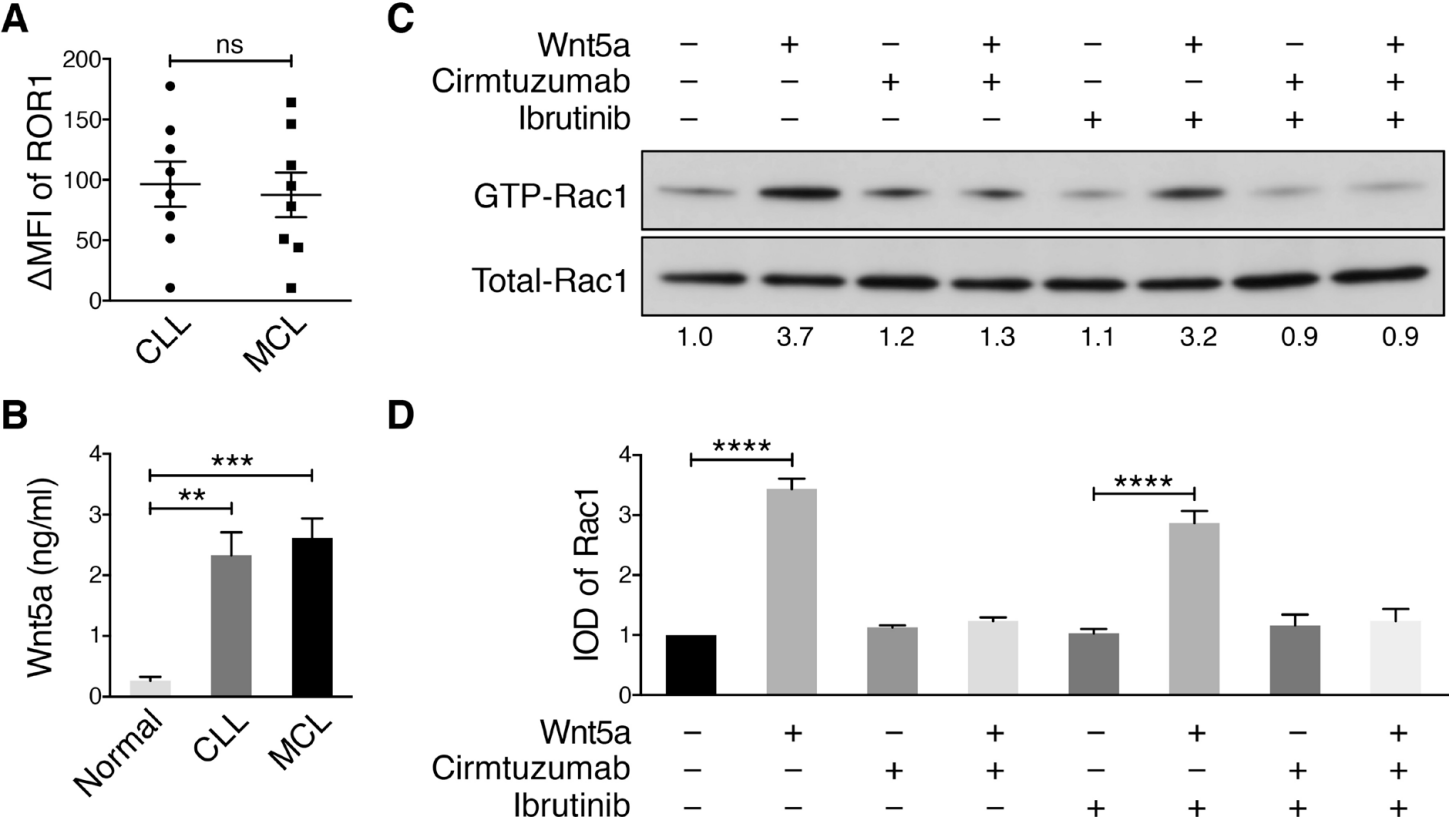
Cirmtuzumab inhibits Wnt5a-Induced Rac1 activation in ibrutinib-treated MCL cells (**A**) DMFI of ROR1 in MCL vs CLL (*n* = 8). The *P* value was determined by Student’s *t* test; ns = not significant. (**B**) Plasma Wnt5a in patients with MCL versus age-matched control subjects (*n* = 4 per group; *P* < 0.05, Student’s *t* test). (**C**) Activated Rac1 was measured in MCL cells, which were treated with or without Wnt5a (200 ng/ml), with or without ibrutinib (0.5 µM), or with or without cirmtuzumab (10 μg/ml), as indicated above each lane of the immunoblot. The numbers below each lane are the ratios of band integrated optical density (IOD) of activated versus total Rac1 normalized to untreated samples (defined as the “IOD of Rac1”). (**D**) Depicts the mean IOD of Rac1, as defined in 1C, in Wnt5a-stimulated MCL cells treated with or without cirmtuzumab and/or ibrutinib observed in three independent experiments (± SEM). ^****^P < 0.0001, as calculated using one-way ANOVA with Tukey’s multiple comparisons test.

We examined for expression of ROR1 by flow cytometry and determined the “ROR1 ∆MFI”, which is the mean fluorescence intensity of CD19^+^ cells stained with an Alexa-647-conjugated anti-ROR1 mAb (4A5) minus the mean fluorescence intensity of the CD19^+^ cells stained with an Alexa-647-conjugated non-specific mAb of the same isotype. We found the median ROR1 ∆MFI of primary MCL cells was similar with that of CLL cells (Figure 1A), which in turn was significantly greater than that of healthy-adult blood B cells, which had undetectable ROR1 [5]. Similar to what we observed in patients with CLL, [1] we also found the plasma of patients with MCL have high levels of Wnt5a relative to that of healthy adults (Figure 1B).

MCL cells were cultured with ibrutinib, cirmtuzumab, or both ibrutinib and cirmtuzumab for 2 h, and then stimulated with exogenous Wnt5a for 30 min. Ibrutinib or cirmtuzumab did not induce apoptosis of MCL cells under these culture conditions, which did not include complement or immune effector cells. For comparison, cells from the same MCL sample were cultured without Wnt5a in parallel. This revealed that Wnt5a induced Rac1 activation in the primary MCL cells and that this effect could be blocked by cirmtuzumab, but not ibrutinib, as observed with primary CLL cells (Figure 1C–1D).

**Figure 2:**
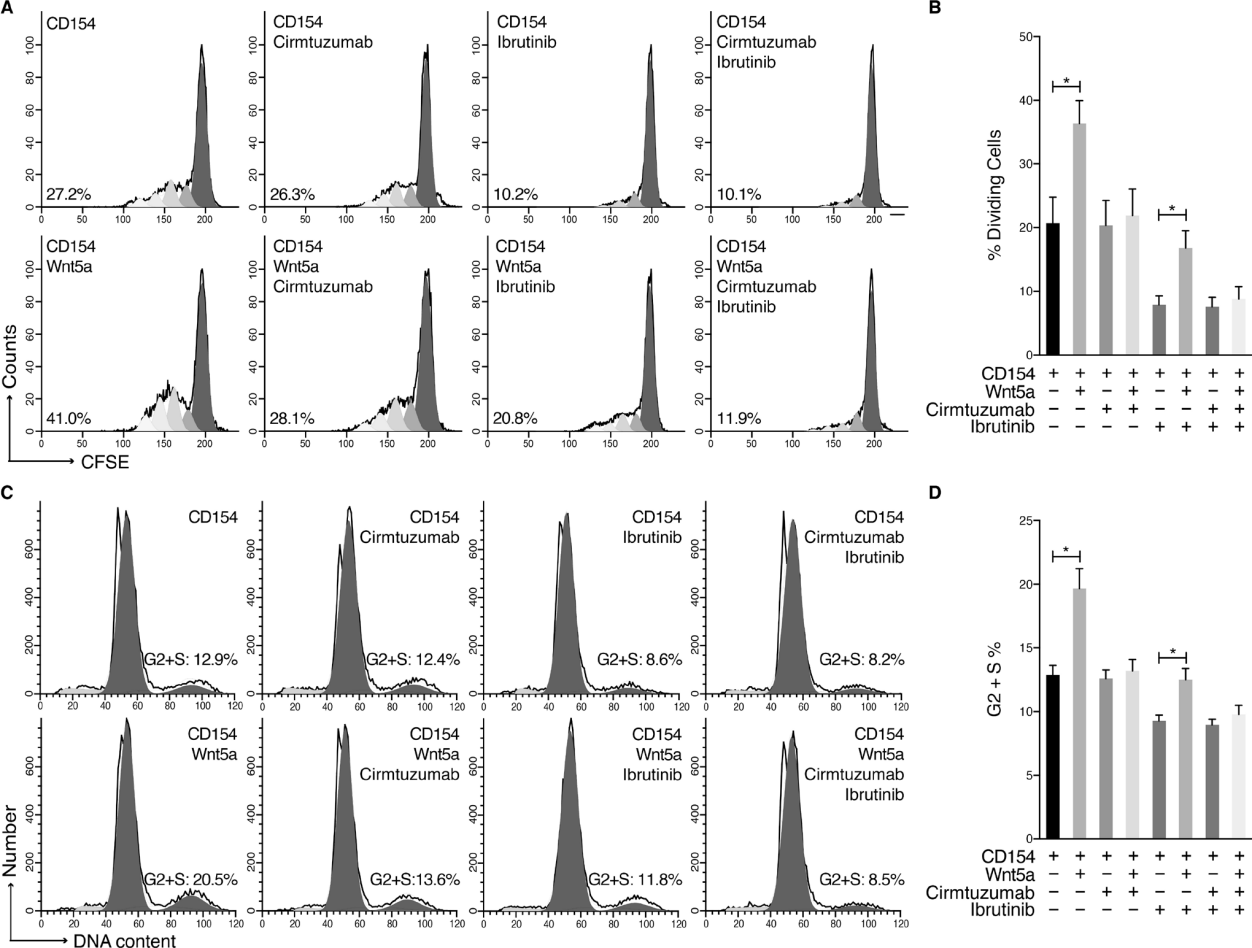
Cirmtuzumab inhibits Wnt5a-enhanced proliferation in ibrutinib-treated MCL Cells (**A**) CD154-induced proliferation of CFSE-labeled MCL cells (*n* = 3) with or without Wnt5a and treated with cirmtuzumab (10 μg/ml) or ibrutinib (0.5 μM). One representative MCL sample is shown with the percent of dividing cells. (**B**) The bars indicate the mean proportions of MCL cells with diminished CFSE fluorescence from each of 3 different patients for each culture condition, as indicated at the bottom. (**C**) MCL cells (*n* = 3) were cultured with or without Wnt5a in the presence of CD154 and IL-4/10, and then treated with cirmtuzumab (10 μg/ml) or ibrutinib (0.5 μM) for 4 days, subjected to cell-cycle analysis following propidium iodide staining. One representative MCL sample is shown. (**D**) The mean fraction of cells in S/G2 phase for all 3 patients tested is presented. Data are shown as mean ± SEM, ^*^P < 0.05, as determined by one-way ANOVA with Tukey’s multiple comparisons test.

We induced proliferation of MCL cells by culturing MCL cells with recombinant interleukin (IL)-4 and IL-10 together with HeLa cells transfected to express CD154 (HeLa_CD154_) [6, 7]. To examine for proliferating cells, we stained the MCL cells with carboxyfluorescein succinimidyl ester (CFSE), allowing us to monitor for cells with lower fluorescence that had undergone cell division. Addition of exogenous Wnt5a to co-cultures of MCL cells on HeLa_CD154_ cells significantly enhanced the proportion of MCL cells undergoing cell division (Figure 2A–2B). Moreover, treatment of the MCL cells with cirmtuzumab, but not ibrutinib, blocked the capacity of Wnt5a to enhance MCL-cell proliferation (Figure 2A–2B). Cell-cycle analysis on permeabilized MCL cells using propidium iodide showed that Wnt5a stimulation significantly enhanced the fraction of CD154-stimulated MCL cells in S/G2 (Figure 2C–2D). The capacity of Wnt5a to enhance the proportion of cells in S/G2 could be inhibited by treatment with cirmtuzumab, but not ibrutinib (Figure 2C–2D). Collectively, these data demonstrate that cirmtuzumab could block Wnt5a-signaling leading to enhanced MCL-cell proliferation, which was not affected by treatment with ibrutinib.

## DISCUSSION

In this study, we examined primary circulating leukemia cells of patients with MCL undergoing treatment with ibrutinib, which is highly effective at inhibiting BCR-signaling through its capacity to inhibit BTK. [8, 9] First, we noted that median ROR1 expression level of primary MCL cells was similar with that of CLL cells and that the plasma of patients with MCL had high levels of Wnt5a that were comparable to those found in patients with CLL; in contrast Wnt5a was virtually undetectable in the plasma of age-matched healthy adults. We found that Wnt5a can induce Rac1 activation in the primary MCL cells, as noted in a variety of cell types, [10–12] including CLL cells, [1] and cirmtuzumab, but not ibrutinib, could inhibit the capacity of Wnt5a to induce primary MCL cells to activate Rac1, which is similar to the findings of Ren and colleagues, who reported that ibrutinib could not inhibit FcγR-induced Rac1 activation, even though it could inhibit FcγR-induced calcium signaling and cytokine production, [13] and our previous reporting that Wnt5a-signaling was dependent upon ROR1, but not ibrutinib, as indicated by the capacity of cirmtuzumab to inhibit Wnt5a-induced activation of Rac1 in CLL cells [2]. We conclude that ibrutinib cannot block ROR1-dependent Wnt5a-induced activation of Rac1, which serves as an intracellular signal transducer that can influence multiple signaling pathways.

Prior studies found that activated Rac1 could enhance resistance of neoplastic cells to cytotoxic drugs. One study found that activated T cells and fibroblasts could induce activation of Rac1 in neoplastic B cells and thereby enhance their resistance to the cytotoxic effects of fludarabine monophosphate; inhibition of activated Rac1, on the other hand, could enhance the sensitivity of neoplastic B cells to the cytotoxic effects of this drug [14]. In another study, Rac1 was found to interact with and enhance the function of Bcl-2 [15]. which could inhibit spontaneous or drug-induced apoptosis. Conversely, another study found that treatment of leukemia B cells with NSC-23766, an inhibitor of activated Rac1, enhanced the capacity of Bcl-2 antagonists to induce apoptosis [16]. Loss of p53 in lymphoma B cells has been associated with increased activation of Rac1, which could be inhibited by NSC-23766 or a dominant-negative form of Rac1, Rac1N17, causing a dose-dependent increase in the rate of spontaneous or drug-induced apoptosis [17]. Furthermore, activation of Rac1-GTPase can enhance proliferation [1, 2, 14], whereas loss of activated Rac1 can result in impaired hematopoietic-cell growth [18]. As such, the activated Rac1 observed in MCL cells may provide an ancillary stimulus, which may enhance the survival of MCL cells of patients treated with ibrutinib, which typically lacks the ability to induce complete responses in most patients treated. Conceivably, concomitant treatment of patients with MCL using cirmtuzumab and ibrutinib could improve the complete response rate to ibrutinib therapy, which yields lower overall response rates and a shorter progression free survival for patients with MCL than for patients with CLL [19–21].

In summary, this study demonstrates that Wnt5a can induce ROR1-dependent activation of Rac1 in MCL and that such signaling could be inhibited by cirmtuzumab. Because ROR1-signaling induces activation of Rac1 via a pathway that cannot be inhibited by ibrutinib, these results support the notion that ibrutinib and cirmtuzumab may have complementary effects in the treatment of patients with MCL. These results are comparable to what we observed with CLL B cells, for which we found cirmtuzumab to have synergistic and complementary anti-tumor activity with ibrutinib in clearing leukemia cells in 3 different animal models [2]. Collectively, these results provide rationale for clinical testing of these drugs in combination to determine the safety and clinical activity of cirmtuzumab and ibrutinib in patients with relapsed or refractory CLL or MCL.

## MATERIALS AND METHODS

### Blood samples

Blood samples were collected from MCL patients at the University of California San Diego Moores Cancer Center who satisfied diagnostic and immunophenotypic criteria for MCL, and who provided written, informed consent, in compliance with the Declaration of Helsinki and the Institutional Review Board (IRB) of the University of California San Diego (IRB approval number 080918). PBMCs, of which >90% were MCL cells, were isolated as described [1].

### Flow cytometry analysis

Flow cytometry analysis was performed as described [22]. The following antibodies were used to stain cells at 4° C for 20 min: anti-ROR1 mAb (4A5) conjugated with Alexa-647 (4A5-Alexa-647) was generated in our laboratory. Anti-CD23 mAb conjugated with FITC (anti-CD23-FITC) was ordered from Life Technologies (Cat #MHCD2301). Anti-CD200 mAb conjugated with PE (anti-CD200-PE) was obtained from BD Biosciences (Cat #552475). PerCP-Cy5.5 conjugated anti-CD19 was purchased from Ebioscience (Cat #45-0199-42) and PE conjugated anti-CD5 antibodies were from BD Biosciences (Cat, #555355). The stained cells were washed twice with FACS buffer (phosphate buffered saline, pH 7.4 (PBS), 3% FBS), and examined by four-color, multiparameter flow cytometry using a dual-laser FACSCalibur (BD Biosciences), and the data were analyzed using FlowJo software (TreeStar). We subtracted the mean-fluorescence intensity (MFI) of cells stained with a fluorochrome-labeled, isotype-control mAb from the MFI of the same cells stained with each antibody to determine the specific increase in MFI (ΔMFI).

### Rac1 activation assay

Rac1 activation assay reagents were purchased from Cytoskeleton and used as per manufacturer’s instruction. Briefly, GTP-bound active Rac1 were pulled down with PAK-PBD beads, and then subjected to immunoblot analysis. Immunoblots of whole-cell lysates were used to assess for total Rac1. The integrated optical density (IOD) of bands was evaluated by densitometry and analyzed using Gel-Pro Analyzer 4.0 software (Media Cybernetics, MD).

### Immunoblot analysis

Western blot analysis was performed as described [22]. Equal amounts of total protein from each sample were fractionated by SDS-PAGE and blotted onto polyvinylidene difluoride membrane. Western blot analysis was performed using primary mAb specific for Rac1, which were detected using secondary antibodies conjugated with horseradish peroxidase (Cell Signaling Technology).

Plasma Wnt5a enzyme-linked immunosorbent assay

Enzyme-linked immunosorbent assay (ELISA) kit (MyBioSource) was used to measure Wnt5a levels in plasma samples from 9 patients with CLL and 9 healthy individuals, as per the manufacturer’s instruction.

### Cell proliferation assay

MCL cells were labeled by carboxyfluorescein succinimidyl ester (CFSE, Life Technologies) and plated at 1.5 × 10^6^/well/ml in a 24-well tray on a layer of irradiated HeLaCD154 cells (8000 Rad; 80 Gray) at a MCL/HeLaCD154 cell ratio of 15:1 in complete RPMI-1640 medium supplemented 5 ng/mL of recombinant human interleukin (IL)-4 (R&D Systems) and 15 ng/mL recombinant human IL-10 (R&D Systems). Wnt5a (200 ng/ml, R&D Systems) or cirmtuzumab (10 μg/ml) as indicated in the text. CFSE-labeled MCL cells were analyzed by flow cytometry; Modfit LT software (version 3.0, Verity Software House) was used for analysis of cell proliferation as previously described44,45.

### Cell cycle analyses

MCL cells (1 × 10^7^) were suspended in 100 µl of PBS and fixed overnight at 4° C by adding 1 ml cold ethanol. Cells were spin at 700 × g for 2 min and washed twice with PBS containing 1% BSA. The pelleted cells were then suspended in 500 µl of PBS containing 1% BSA and 1 µl of RNase (100 mg/ml); RNase was added to digest RNA. PI solution (0.5 mg/ml in 38 mM sodium citrate, pH 7.0), 1 µl boiled RNase A (100 mg/ml), and PI-staining solution (0.5 mg/ml in 38 mM sodium citrate, pH 7.0; 60 µl) were added to cells and incubated in the dark for 1 hour at room temperature. Immediately thereafter, the cells were analyzed via flow cytometry using a FACSArray (Becton Dickinson), and data were analyzed using FlowJo software (Tree Star Inc.).

### Statistics

Data are presented as mean ± SEM as indicated, for data sets that satisfied conditions for a normal distribution, as determined by the Kolmogorov-Smirnov test. Differences between two groups were determined by unpaired 2-tailed Student’s *t* test. The statistical significance of the difference between means was assessed by one-way ANOVA with Tukey’s multiple comparisons test. *P* values less than 0.05 were considered significant. Analysis for significance was performed with GraphPad Prism 6.0 (GraphPad Software Inc.).

## SUPPLEMENTARY MATERIALS FIGURE


