# Supplementary material for: Cirmtuzumab inhibits ibrutinib-resistant, Wnt5a-induced Rac1 activation and proliferation in mantle cell lymphoma
Source: Oncotarget. 2018 May 15;9(37):24731–6. doi: 10.18632/oncotarget.25340 (PMC5973864; doi:10.18632/oncotarget.25340)
Supplement: Supplementary file 1 [file oncotarget-09-24731-s001.pdf]

Cirmtuzumab inhibits ibrutinib-resistant, Wnt5a-induced Rac1 activation and proliferation in mantle cell lymphoma

SUPPLEMENTARY MATERIALS

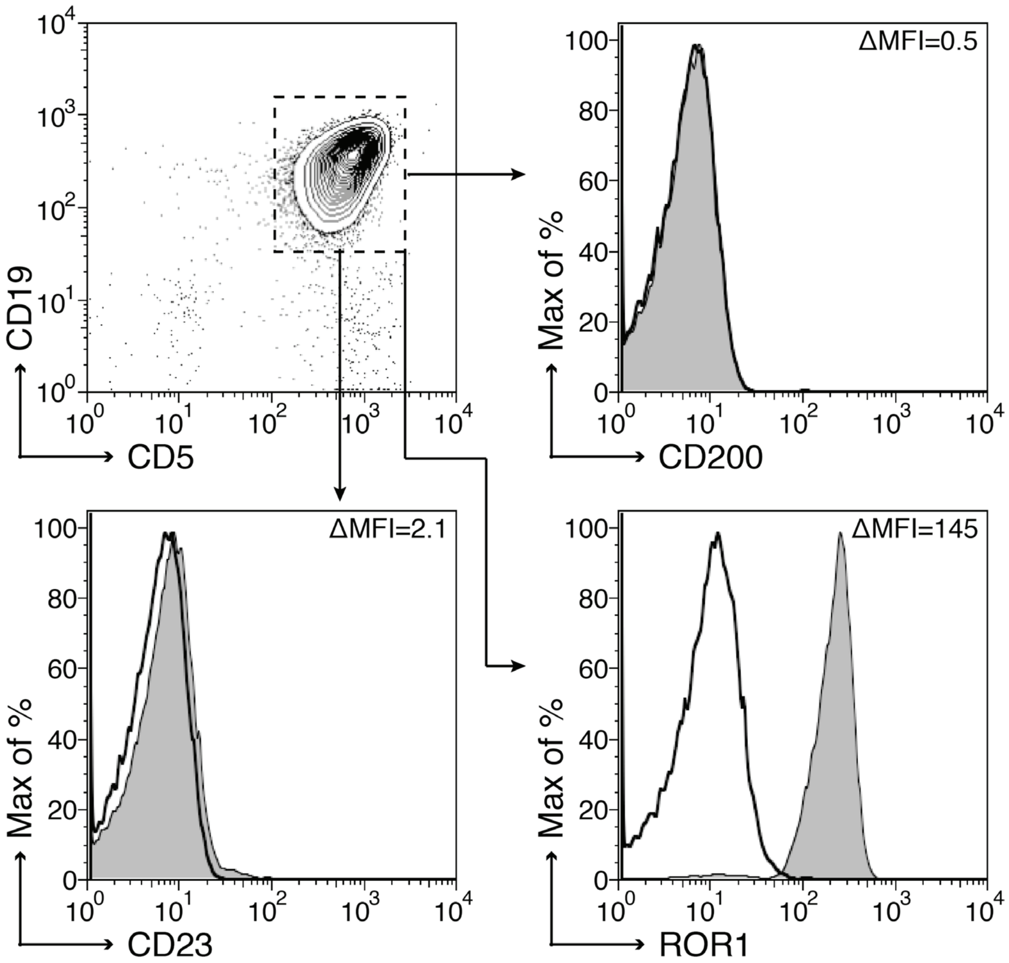

Supplementary Figure 1: Surface antigen expression on MCL cells. Gating on the MCL cells, which express CD5 and CD19 (top left). The shaded histograms show the fluorescence of the gated MCL cells stained with fluorochrome-conjugated mAb specific for other surface antigens. In contrast to CLL cells, the MCL cells failed to stain with a mAb specific for CD200 (top right) or CD23 (bottom left). MCL cells typically express high levels of ROR1 (bottom right). The open histograms depict fluorescence of cells stained with an isotype control antibody.
